# A Functional Variant of the Dimethylarginine Dimethylaminohydrolase-2 Gene Is Associated with Insulin Sensitivity

**DOI:** 10.1371/journal.pone.0036224

**Published:** 2012-04-27

**Authors:** Francesco Andreozzi, Ivan Presta, Gaia Chiara Mannino, Daniela Scarpelli, Sara Di Silvestre, Natalia Di Pietro, Elena Succurro, Angela Sciacqua, Assunta Pandolfi, Agostino Consoli, Marta Letizia Hribal, Francesco Perticone, Giorgio Sesti

**Affiliations:** 1 Department of Medical and Surgical Sciences, University “Magna Græcia" of Catanzaro, Catanzaro, Italy; 2 Department of Biomedical Sciences, University “G. d'Annunzio", Chieti-Pescara, Italy; 3 Department of Medicine and Aging Sciences, University “G. d'Annunzio", Chieti-Pescara, Italy; 4 Aging Research Center (Ce.S.I.), “G. d'Annunzio" University Foundation, Chieti-Pescara, Italy; University of Padova, Italy

## Abstract

**Background:**

Asymmetric dimethylarginine (ADMA) is an endogenous inhibitor of endothelial nitric oxide synthase, which was associated with insulin resistance. Dimethylarginine dimethylaminohydrolase (DDAH) is the major determinant of plasma ADMA. Examining data from the DIAGRAM+ (Diabetes Genetics Replication And Meta-analysis), we identified a variant (rs9267551) in the *DDAH2* gene nominally associated with type 2 diabetes (*P* = 3×10^−5^).

**Methodology/Principal Findings:**

initially, we assessed the functional impact of rs9267551 in human endothelial cells (HUVECs), observing that the G allele had a lower transcriptional activity resulting in reduced expression of DDAH2 and decreased NO production in primary HUVECs naturally carrying it. We then proceeded to investigate whether this variant is associated with insulin sensitivity *in vivo*. To this end, two cohorts of nondiabetic subjects of European ancestry were studied. In sample 1 (n = 958) insulin sensitivity was determined by the insulin sensitivity index (ISI), while in sample 2 (n = 527) it was measured with a euglycemic-hyperinsulinemic clamp. In sample 1, carriers of the GG genotype had lower ISI than carriers of the C allele (67±33 vs.79±44; *P* = 0.003 after adjusting for age, gender, and BMI). ADMA levels were higher in subjects carrying the GG genotype than in carriers of the C allele (0.68±0.14 vs. 0.57±0.14 µmol/l; *P* = 0.04). In sample 2, glucose disposal was lower in GG carriers as compared with C carriers (9.3±4.1 vs. 11.0±4.2 mg×Kg^−1^ free fat mass×min^−1^; *P* = 0.009).

**Conclusions/Significance:**

A functional polymorphism of the *DDAH2* gene may confer increased risk for type 2 diabetes by affecting insulin sensitivity throughout increased ADMA levels.

## Introduction

Endothelial dysfunction is thought to be an early abnormality in the development and progression of atherosclerosis [1], [2]. Several lines of evidences suggest that endothelial dysfunction is associated with insulin-resistance states, including obesity, dyslipidemia, and hypertension [3]–[6]. Furthermore, it has been reported that endothelial dysfunction predicts type 2 diabetes mellitus independently from other well-known diabetes risk factors [7]–[9]. Endothelial dysfunction is a broad term that implies reduced production and/or availability of nitric oxide (NO). Inhibition of NO synthesis by endogenous inhibitors of the endothelial NO synthase (eNOS) may play an important role in inducing endothelial dysfunction [10]. A decreased NO bioavailability may also have a significant impact on the development of insulin resistance; NO modulates insulin-mediated glucose disposal as well as vessels reactivity in insulin-sensitive tissue such as the skeletal muscle [11]. It has been shown that changes in insulin-mediated capillary recruitment are positively correlated with changes in insulin-stimulated glucose disposal [12]. Moreover, mice with a genetic disruption of endothelial NOS exhibit insulin resistance caused by reduction in both microvascular recruitment and muscle glucose uptake in response to insulin [13]. Asymmetric dimethylarginine (ADMA) is a naturally occurring methylated arginine that inhibits the synthesis of NO from L-arginine thus impairing endothelium-dependent vasodilation [14], [15]. Elevated plasma levels of ADMA have been reported in several clinical syndromes associated with increased cardiovascular disease risk, including type 2 diabetes [16]–[21], and have been associated with insulin resistance [22]–[24]. There is evidence that treatment with rosiglitazone, an insulin sensitizer, improves insulin sensitivity and reduces plasma ADMA concentration in insulin-resistant subjects with hypertension [22]. ADMA is generated by methylation of proteins by the protein arginine N-methyltransferases (PRMTs), and is metabolised by the dimethylarginine dimethylaminohydrolase (DDAH) [25], [26]. Two isoforms of DDAH have been identified, with DDAH1 found in tissues expressing neuronal NOS, and DDAH2 highly expressed in the endothelium [27]. Transgenic mice over-expressing DDAH1 exhibit reduced ADMA levels, which are associated with an increased production of NO, and enhanced insulin sensitivity [28], [29]. At the molecular level, transgenic mice over-expressing DDAH1 show an enhanced hepatic Akt phosphorylation. Exogenous administration of ADMA to normal mice results in an impaired insulin-stimulated glucose incorporation into glycogen in skeletal muscle; these data support the hypothesis that ADMA may modulate insulin sensitivity, by decreasing NO synthesis [29].

In view of the important role of ADMA in regulating endothelium-dependent vasodilation and, thereby, insulin sensitivity, we hypothesized that polymorphisms in the *DDAH2* gene, potentially altering its expression or activity, may be associated with type 2 diabetes. Therefore, we interrogated the Diabetes Genetics Replication And Meta-analysis (DIAGRAM+) Consortium database [30] to investigate if the three polymorphisms potentially affecting *DDAH2* gene expression were associated with type 2 diabetes. One variant (rs9267551) in the *DDAH2* gene was nominally associated with type 2 diabetes (*P* = 3×10^−5^), although this association did not reach genome-wide significance (*P*<5×10^−8^), with the G diabetogenic risk allele conferring an odds ratio (OR) of 1.12 (95% CI 1.06–1.19) (Andrew Morris and Mark McCarthy personal communication for the DIAGRAM+). The two other variants (rs805305 and rs707916) in perfect linkage disequilibrium (r^2^ = 1.0), supposed to have functional effects [31], [32] on ADMA levels, were not associated with type 2 diabetes (OR = 1.02, 95% CI 0.98–1.07) (Andrew Morris and Mark McCarthy personal communication for the DIAGRAM+). Since rs9267551 variant is located in a regulatory region of *DDAH2* comprising consensus binding motifs sequences recognized by Egr and EF2 trancription factors families [33], we performed a functional analysis in endothelial cells and found that the rs9267551 polymorphism can act as a modulator of mRNA expression of *DDAH2*, and, then, investigated whether rs9267551 genotypes were associated with insulin sensitivity in two independent samples of nondiabetic subjects of European ancestry.

## Materials and Methods

### Cell cultures

Umbilical cords were obtained from randomly selected healthy mothers delivering at the Pescara Town Hospital who had signed a written consent form. Umbilical cord arteries were screened for the polymorphism of interest as described below. Primary HUVECs were obtained as previously described [34], [35] and cultured in EGM-2 medium (Cambrex Bioscience, East Rutherford, NJ) supplemented with 2% Fetal Bovine Serum, ascorbic acid, VEGF, rhIGF-1, hydrocortisone, rhFGF, rhEGF, heparin, amphotericin B and penicillin/streptomycin. We did not find HUVECs carrying the C/C genotype since the proportion of individuals carrying this genotype is very low (<1% in the two study samples). The immortalized HUVECs cell line was obtained from Cambrex Bioscience (East Rutherford, NJ) and maintained in EGM-2 medium.

### Constructs preparation and luciferase assay

A 1755 bp fragment of the human *DDAH2* gene spanning the promoter region and the first exon, was obtained by polymerase chain reaction (PCR) from a human genomic DNA sample and the variant carrying the rs9267551 C minor allele was generated using the QuickChange Site-Directed Mutagenesis kit (Stratagene, Las Vegas, NV). Both fragments were inserted in the pGL3 basic luciferase expression vector (Promega, Madison, WI) and constructs sequence was confirmed by direct sequencing. HUVECs were seeded on 12-wells plates cotransfected with 0.8 µg of either pGL3 rs9267551G, pGL3 rs9267551C or an empty pGL3 vector, and 70 ng phRL-Renilla vector, as an internal control of transfection efficiency, per well, using lipofectamine transfection reagents (Invitrogen Co., Carlsbad, CA), according to the manufacturer protocol. Luciferase activities were determined 24 h after transfection, using the dual luciferase reporter assay system with a 20/20 luminometer (Promega, Madison, WI).

### RNA extraction and Real Time PCR

Total RNA was obtained from primary HUVECs carrying the different genotypes (G/G and C/G), reverse transcribed using the High Capacity cDNA Reverse Transcription Kit (Applied Biosystems, Foster City, CA) and analyzed by Real-Time quantitative PCR using a Power SYBR Green PCR Master Mix (Applied Biosystems, Foster City, CA) with 10 ng of cDNA and 0.2 µM of the following oligonucleotide primers: FW: 5′-GGTGGTGGGAGGTAAACTGA-3′; RV: 5′-TCGCGTTCTCGTCTCCTATT-3′. The human ribosomal protein S9 (RPS9) was amplified using the following oligonucleotide primers: FW: 5′-CTGGGTTTGTCGCAAAACTT-3′; RV: 5′-GTGGGTCCTTCTCATCAAGC-3′, and used to normalize the results according to the Livak method.

### Nitric oxide synthase (NOS) activity in HUVECs

HUVECs were starved for 12 h in serum-deprived medium and NOS activity was determined in duplicate for each cell strain by measuring the conversion of L-(3H)-arginine into L-(3H)-citrulline as previously described [34], [35]. Data of NOS activity were normalized to cell protein content.

### DNA analysis

DNA was isolated from whole blood using commercial DNA isolation kit (Promega, Madison, WI). Screening of the rs9267551 polymorphism was performed using a TaqMan allelic discrimination assay (Applied Biosystems, Foster City, CA). TaqMan genotyping reaction was amplified on a GeneAmp PCR system 2700 and fluorescence was detected using an ABI Prism 7000 sequence detector (Applied Biosystems, Foster City, CA). Genotyping quality was tested by including 12 HapMap samples in each 384-well assay. The agreement rate with the HapMap database genotypes was >99%.

### Study subjects

Two different samples of adult (≥18 years of age) nondiabetic Italians of European ancestry were studied. Diabetes mellitus was defined as fasting plasma glucose >126 mg/dl, current treatment with anti-diabetic drugs or self-reported history of a previous diagnosis.

Sample 1 comprised 958 nondiabetic individuals (438 men and 520 women, age 51.0±12.9 years, BMI 30.6±6.4 kg/m2), consecutively recruited at the Department of Experimental and Clinical Medicine of the University ‘Magna Graecia’ of Catanzaro [36]. All individuals underwent an oral glucose tolerance test (OGTT; 75 g) with glucose and insulin levels measured before and after glucose load. The Matsuda index [insulin sensitivity index (ISI)] was calculated as follows: 10,000/square root of [fasting glucose (millimoles per liter)×fasting insulin (milliunits per liter)]×[mean glucose×mean insulin during OGTT]. ADMA concentrations in plasma were measured in 68 subjects by high-performance liquid chromatography, as previously described [15].

Sample 2 comprised 527 non-diabetic offspring of patients with type 2 diabetes (228 men and 299 women, age 39.1±10.5 years, BMI 29.2±6.3 kg/m2) from the EUGENE2 project (http://www.eugene2.com), consecutively recruited at the Department of Experimental and Clinical Medicine of the University ‘Magna Graecia’ of Catanzaro according to previously reported inclusion criteria [37]. After 12-h overnight fast, subjects underwent a euglycemic hyperinsulinemic clamp study as previously described [38], [39]. The rate of total insulin-stimulated glucose disposal (M) was calculated for the last 60 minutes of the insulin infusion, and normalized per kg of fat-free mass.

All the investigations were performed in accordance with the principles of the Declaration of Helsinki, and the protocol was approved by the local ethical committee. All subjects recruited provided written informed consent.

### Statistical analysis

The results for continuous variables are given as means ± SD. Unpaired Student's t test or ANOVA were used to compare differences of continuous variables between groups, as appropriate and the χ2-test for non-continuous variables. A general linear model was used to compare phenotypic differences in the two samples. Power calculations were performed with Quanto version 1.2.4 (http://hydra.usc.edu/gxe; accessed 25 July 2011). The study had 80% power (for α = 0.05) to detect a 15% increase in insulin sensitivity per copy of allele C in sample 1 and a 17% increase in insulin sensitivity per copy of allele C in sample 2. Associations between polymorphism and continuous traits are presented as effect sizes (β and SE) per copy of the type 2 diabetes risk allele, estimated by linear regression adjusted for gender, age, and BMI. We report nominal *P* value<0.05 without adjustment for multiple testing given the high prior probabilities for association of the rs9267551 polymorphism with the examined phenotype. All analyses were performed using the SPSS software program Version 16.0 for Windows.

## Results

**Figure 1:**
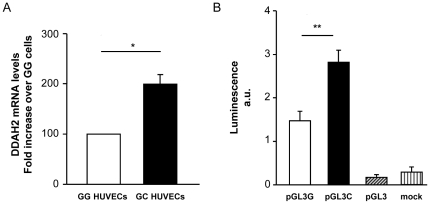
Functional effects of the rs9267551 polymorphism. **A.** Quantification of DDAH2 mRNA levels in primary HUVECs naturally carrying the G/G (n = 10) or C/G genotype (n = 5). Total RNA was extracted from confluent cells. cDNA was reverse transcripted and analyzed by quantitative Real-Time PCR technique. Bars represent the means ± SD of two independent experiments carried out in triplicate (*P = 0.008 for G/G HUVECs vs C/G HUVECs); **B.** Luciferase activity was evaluated in immortalized HUVEC transfected with an empty PGL3 luciferase vector (PGL3, light gray bar, n = 21); or a PGL3 vector carrying the rs9267551 G (PGL3G, white bar, n = 21); or a PGL3 vector carrying the rs9267551 C form (PGL3C, black bar, n = 21). Luciferase activity in mock transfected cells (mock, dark gray bar, n = 21) was assessed as a control. (**P = 0.00076 for PGL3G vs. PGL3C).

**Figure 2:**
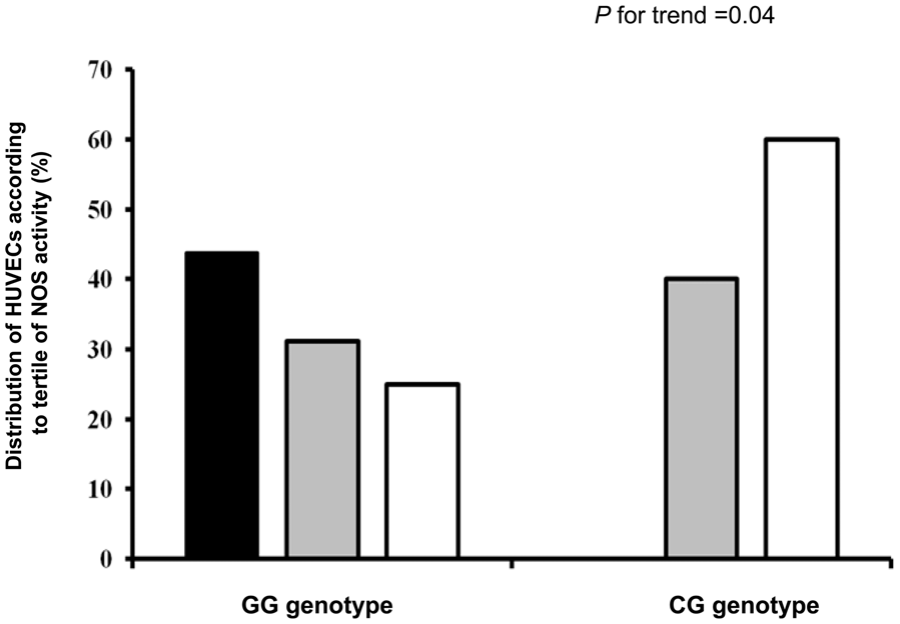
Stratification of HUVEC strains according to tertiles of NOS activity (%). The proportion of HUVECs displaying a lower NO release was significantly higher among cells carrying the GG genotype (P for trend = 0.04).

Initially, to evaluate the hypothesis that rs9267551 polymorphism may affect *DDAH2* transcription, we assessed DDAH2 mRNA levels in primary HUVECs naturally carrying the rs9267551 GG and CG genotype by Real-Time RT-PCR. We observed that *DDAH2* expression was significantly lower in GG HUVECs as compared to CG cells (*P* = 0.008; Fig. 1A). Subsequently, to confirm a direct effect of rs9267551 on *DDAH2* transcription levels, we transfected immortalized HUVEC cells with a luciferase expression vector carrying either the rs9267551 C or the rs9267551 G allele and evaluated the transcription efficiency of the luciferase gene 24 hrs after transfection. We observed that the G allele was associated with a significant decrease in luciferase activity as compared to the C allele (*P* = 0.00076; Fig. 1B). Finally, we tested whether rs9267551 polymorphism affects NOS activity, as evaluated by assessing the conversion of L-[3H]arginine into L-[3H] citrulline in HUVECs carrying either the GG (n = 34) or the CG (n = 5) genotype. In the presence of 1 mM arginine, basal nitric oxide release was lower in GG HUVECs as compared to CG cells (0.145±0.08 and 0.198±0.19 pmoles/NO/min/mg protein, respectively) although these differences did not reach the significance threshold (*P* = 0.08). By stratifying cell strains according to tertiles of NOS activity, the proportion of HUVECs displaying a lower NO release was significantly higher among cells carrying the GG genotype (*P* for trend = 0.04) (Fig. 2).

Next, we tested the hypothesis that the rs9267551 polymorphism was associated with insulin sensitivity in two samples of nondiabetic individuals. In sample 1 insulin sensitivity was assessed by the ISI index (calculated from glucose and insulin levels at 0, 30, and 120 min of the OGTT) whereas in sample 2 insulin sensitivity was directly measured by the euglycaemic-hyperinsulinemic clamp, considered as a reference assay for insulin sensitivity. It is noteworthy that among surrogate indexes of insulin sensitivity, the ISI index has been showed to have the highest correlation with insulin-stimulated glucose disposal measured by the clamp-derived index [40], [41].

Clinical characteristics of sample 1 and sample 2 according to the rs9267551 polymorphism are shown in Table 1. Genotype distributions were in Hardy-Weinberg equilibrium (*P*>0.05) in each study sample. Because of the small number of CC individuals (n = 5) and the a priori hypothesis based on the dominant effect observed in functional studies in endothelial cells, GC and CC individuals were pooled and analyzed together as C carriers, according to a dominant genetic model. The rs9267551 polymorphism did not show any significant association with age, gender, and body mass index (BMI) (Table 1), whereas it was significantly associated with insulin sensitivity, as estimated by the ISI index, with subjects carrying the GG genotype having significantly lower insulin sensitivity as compared with carriers of the C allele (67±33 vs. 79±44, respectively; *P* = 0.003 after adjusting for age, gender, and BMI) (Table 1). ADMA concentrations in plasma were available for 60 subjects carrying the GG genotype, and 8 carriers of the C allele (7 subjects carrying the GC genotype and 1 subject carrying the CC genotype). ADMA levels were significantly higher in subjects carrying the GG genotype as compared with carriers of the C allele (0.68±0.14 vs. 0.57±0.14 µmol/l, respectively; *P* = 0.04 after adjusting for age, gender, and BMI).

**Table 1:** Clinical features of 958 study subjects (sample 1) and of 527 study subjects (sample 2) according to the rs9267551 polymorphism of *DDAH2*.

	Variables	GG	GC+CC	*P*	*β*	SE	*P*
**Sample 1**	Male/Female	404/475	34/45	0.61			
	Age (*yrs.*)	51±12	50±14	0.59§			
	BMI (*kg/m^2^*)	30.5±6.3	30.9±6.6	0.65#			
	ISI	67±33	79±45	0.003*	−11.63	3.54	0.001
**Sample 2**	Male/Female	210/265	18/34	0.18			
	Age (*yrs.*)	39±10	40±11	0.24§			
	BMI (*kg/m^2^*)	29.2±6.2	28.3±5.9	0.17#			
	Insulin-stimulated glucose disposal (*mg×Kg^−1^ FFM×min^−1^*)	9.3±4.1	11.0±4.2	0.03*	−1.09	0.55	0.04

Data are means ± SD. Comparisons between two groups were performed using unpaired Student's t. Categorical variables were compared by χ^2^ test.

§ *P* values refer to results after analyses with adjustment for gender.

# *P* values refer to results after analyses with adjustment for age, and gender using a general linear model.

* *P* values refer to results after analyses with adjustment for age, gender and BMI using a general linear model. Effect sizes (β and SE) per type 2 diabetes risk allele and corresponding *P* values are shown. ISI = insulin sensitivity index.

In order to get further insights on the role of the rs9267551 polymorphism on insulin sensitivity, an additional sample of individuals who underwent a euglycaemic-hyperinsulinemic clamp assay was analysed. Insulin sensitivity assessed as insulin-stimulated glucose disposal was significantly lower in subjects carrying the GG genotype as compared with carriers of the C allele (9.3±4.1 vs. 11.0±4.2 mg×Kg^−1^ free fat mass×min^−1^, respectively; *P* = 0.03 after adjusting for age, gender, and BMI) (Table 1).

## Discussion

Endothelial dysfunction has been associated with insulin resistance and incident type 2 diabetes [3]–[9]. ADMA is an endogenous competitive inhibitor of eNOS and its elevation may account for reduced NO generation observed in states of insulin resistance [22]–[24]. Plasma ADMA levels have been shown to be elevated in patients with type 2 diabetes [21], and have been associated with insulin resistance [22]–[24]. Metabolism of ADMA by DDAH is most likely the major determinant of plasma ADMA concentration [25], [26]. Examining data from the DIAGRAM+ meta-analysis for genes involved in endothelial function led us to the identification of a variant (rs9267551) in the *DDAH2* gene nominally associated with type 2 diabetes (*P* = 3×10^−5^). To find further support for the role of *DDAH2* in the pathogenesis of type 2 diabetes, we examined the functional impact of this polymorphism in human endothelial cells. We observed that the G allele had a lower transcriptional activity resulting in reduced expression of *DDAH2* in primary HUVECs naturally carrying the GG genotype. Therefore, considering the down-regulation of *DDAH2*, a prediction of our in vitro study was that plasma ADMA levels would be higher in carriers of the GG genotype. To verify this conjecture, we measured circulating ADMA concentrations in 68 subjects for whom plasma samples were available. According to our hypothesis, individuals carrying the GG genotype exhibited ADMA levels significantly higher than carriers of the C allele. Importantly, these data are consistent with those recently reported showing that the TGCCCAGGAG haplotype of *DDAH2*, including the G variant of rs9267551, is associated with increased ADMA levels (*P* = 0.0012) [42].

Glucose uptake and metabolism in the skeletal muscle accounts for almost 90% of whole-body glucose disposal in humans [43]; endothelium-derived NO mediates insulin-induced stimulation of skeletal muscle perfusion, and there is evidence that the stimulation of muscle blood flow by insulin promotes substrate delivery and thereby may regulate insulin sensitivity in the skeletal muscle [44]. Accordingly, it has been shown that eNOS knockout mice exhibit a 40% lower insulin-stimulated glucose uptake than control mice as assessed by hyperinsulinemic euglycemic clamp assay [13]. Given the strong association between insulin sensitivity and endothelial function, we next examined the impact of the rs9267551 polymorphism on in vivo insulin sensitivity in two samples of nondiabetic individuals. We found that, as compared with carriers of the C allele, individuals carrying the GG genotype exhibited significantly lower insulin sensitivity estimated by the OGTT-derived ISI, an index of muscle insulin sensitivity strongly correlated with insulin-stimulated total glucose disposal during the euglycemic clamp [45]. These results were confirmed in a second independent sample in which insulin sensitivity was measured by the gold-standard technique, the euglycaemic–hyperinsulinaemic clamp. Interestingly, we did not observe any association between the rs9267551 polymorphism in the *DDAH2* gene and the HOMA index of hepatic insulin resistance (data not shown). These results are consistent with those reported by Stuhlinger et al. [22] showing a significant correlation of plasma ADMA levels with direct measures of insulin-mediated glucose disposal, but not with fasting plasma glucose and insulin levels (the two variables that are used to calculate the HOMA index) in a cohort of nondiabetic individuals. Taken together, these findings suggest that the rs9267551 polymorphism in the *DDAH2* gene through its effects on ADMA levels may impair eNOS activity and NO production resulting in an impaired insulin-stimulated glucose disposal predominantly in skeletal muscle. Our observation that GG HUVECs show a decreased basal NO production offers experimental support to this hypothesis.

The relative homogeneity of study samples with all individuals being Whites of European ancestry represents a strength of this study. An additional strength is constituted by the functional “in vitro" data obtained in human endothelial cells. As a matter of fact, HUVECs are a uniquely suited model to demonstrate a direct effect of a *DDAH2* genetic variant in human endothelium; in fact the in vitro data reported in the study shed light on the molecular mechanism of the association between *DDAH2* and type 2 diabetes observed in the DIAGRAM+ meta-analysis. Indeed, once a putative causal variant has been identified by GWA studies, there is the need to obtain functional confirmation that it is truly causal, and to reconstruct the molecular and physiological mechanisms underlying its impact on the phenotype of interest. Our study, showing the direct influence of the rs9267551 variant on ADMA levels and endothelial function, provides one of the first examples of a SNP identified in GWAs that has a clear functional impact relevant to mechanisms of the disease.

Nonetheless, this study has some limitations. The present findings obtained in a cross-sectional study of cohorts of European ancestry are explorative in nature and replication in independent prospective population-based studies is needed to firmly determine whether the rs9267551 polymorphism affects ADMA levels and insulin sensitivity. A second limitation is that although the results were replicated in two independent samples of Whites of European ancestry, they may not apply to populations of different ethnicity. Another limitation is represented by the very small number of carriers of the C allele of the rs9267551 variant; our finding implies that since the at-risk GG genotype is found in the majority of the general population, *DDAH2* gene variant(s) have a minor role in the genetic susceptibility to insulin-resistance. This minor role is indeed compatible with the fact that the association of *DDAH2* gene variants with type 2 diabetes did not reach genome-wide significance in previous studies. Finally, a major problem in genetic association studies of complex disease is the inconsistency of replication due to small sample size. However, it is worthy to note that: 1) the consistency of the results observed both as risk of type 2 diabetes in the DIAGRAM+ meta-analysis and as intermediate phenotype such as insulin resistance argues against a chance finding; 2) the hypotheses in this study were defined a priori, are biologically plausible and the “in vitro" results are consistent with the “in vivo" data. We therefore believe that our findings are likely to be true.

In conclusion, our data indicate that a functional polymorphism in the *DDAH2* gene may confer increased risk of type 2 diabetes by affecting insulin sensitivity due to an increase in circulating ADMA levels. Although the present findings cannot be presently claimed as definitive, they have to be considered as hypotheses generating and should be replicated in further investigations.

## References

[1] Quyyumi A (1998). Endothelial function in health and disease: new insights into the genesis of cardiovascular disease.. Am J Med.

[2] Panza JA, Quyyumi AA, Brush JE, Epstein SE (1990). Abnormal endothelium dependent vascular relaxation in patients with essential hypertension.. N Engl J Med.

[3] Pinkney JH, Stehouwer CD, Coppack SW, Yudkin JS (1997). Endothelial dysfunction: cause of the insulin resistance syndrome.. Diabetes.

[4] Sciacqua A, Candigliota M, Ceravolo R, Scozzafava A, Sinopoli F (2003). Weight loss in combination with physical activity improves endothelial dysfunction in human obesity.. Diabetes Care.

[5] Ceravolo R, Maio R, Pujia A, Sciacqua A, Ventura G (2003). Pulse pressure and endothelial dysfunction in never-treated hypertensive patients.. J Am Coll Cardiol.

[6] Meigs JB, Hu FB, Rifai N, Manson JE (2004). Biomarkers of endothelial dysfunction and risk of type 2 diabetes mellitus.. JAMA.

[7] Rossi R, Cioni E, Nuzzo A, Origliani G, Modena MG (2005). Endothelial-dependent vasodilation and incidence of type 2 diabetes in a population of healthy postmenopausal women.. Diabetes Care.

[8] Thorand B, Baumert J, Chambless L, Meisinger C, Kolb H (2006). Elevated markers of endothelial dysfunction predict type 2 diabetes mellitus in middle-aged men and women from the general population.. Arterioscler Thromb Vasc Biol.

[9] Perticone F, Maio R, Sciacqua A, Andreozzi F, Iemma G (2008). Endothelial Dysfunction and CRP are risk factors for diabetes in essential hypertension.. Diabetes.

[10] Vallance P, Leiper J (2004). Cardiovascular biology of the asymmetric dimethylarginine:dimethylarginine dimethylaminohydrolase pathway.. Arterioscler Thromb Vasc Biol.

[11] Baron AD (1999). Vascular reactivity.. Am J Cardiol.

[12] Vincent MA, Clerk LH, Lindner JR, Klibanov AL, Clark MG (2004). Microvascular recruitment is an early insulin effect that regulates skeletal muscle glucose uptake in vivo.. Diabetes.

[13] Duplain H, Burcelin R, Sartori C, Cook S, Egli M (2001). Insulin resistance, hyperlipidemia, and hypertension in mice lacking endothelial nitric oxide synthase.. Circulation.

[14] Vallance P, Leone A, Calver A, Collier J, Moncada S (1992). Accumulation of an endogenous inhibitor of nitric oxide synthesis in chronic renal failure.. Lancet.

[15] Perticone F, Sciacqua A, Maio R, Perticone M, Maas R (2005). Asymmetric dimethylarginine, L-arginine, and endothelial dysfunction in essential hypertension.. J Am Coll Cardiol.

[16] Boger RH, Bode-Boger SM, Thiele W, Junker W, Alexander K (1997). Biochemical evidence for impaired nitric oxide synthesis in patients with peripheral arterial occlusive disease.. Circulation.

[17] Kielstein JT, Boger RH, Bode-Boger SM, Schaffer J, Barbey M (1999). Asymmetric dimethylarginine plasma concentrations differ in patients with end-stage renal disease: relationship to treatment method and atherosclerotic disease.. J Am Soc Nephrol.

[18] Miyazaki H, Matsuoka H, Cooke JP, Usui M, Ueda S (1999). Endogenous nitric oxide synthase inhibitor: a novel marker of atherosclerosis.. Circulation.

[19] Valkonen VP, Paiva H, Salonen JT, Lakka TA, Lehtimaki T (2001). Risk of acute coronary events and serum concentration of asymmetrical dimethylarginine.. Lancet.

[20] Zoccali C, Bode-Boger S, Mallamaci F, Benedetto F, Tripepi G (2001). Plasma concentration of asymmetrical dimethylarginine and mortality in patients with end-stage renal disease: a prospective study.. Lancet.

[21] Abbasi F, Asagmi T, Cooke JP, Lamendola C, McLaughlin T (2001). Plasma concentrations of asymmetric dimethylarginine are increased in patients with type 2 diabetes mellitus.. Am J Cardiol.

[22] Stuhlinger MC, Abbasi F, Chu JW, Lamendola C, McLaughlin TL (2002). Relationship between insulin resistance and an endogenous nitric oxide synthase inhibitor.. JAMA.

[23] McLaughlin T, Stühlinger M, Lamendola C, Abbasi F, Bialek J (2006). Plasma asymmetric dimethylarginine concentrations are elevated in obese insulin-resistant women and fall with weight loss.. J Clin Endocrinol Metab.

[24] Perticone F, Sciacqua A, Maio R, Perticone M, Galiano Leone G (2010). Endothelial dysfunction, ADMA and insulin resistance in essential hypertension.. Int J Cardiol.

[25] Ogawa T, Kimono M, Watanabe H, Sasaoka K (1987). Metabolism of NG,NG-dimethylarginine and NG,N′G-dimethylarginine in rats.. Arch Biochem Biophys.

[26] Ogawa T, Kimono M, Watanabe H, Sasaoka K (1989). Purification and properties of a new enzyme NG,NG-dimethylarginine dimethylaminohydrolase from rat kidney.. J Biol Chem.

[27] Leiper JM, Santa Maria J, Chubb A, MacAllister RJ, Charles IG (1999). Identification of two human dimethylarginine dimethylaminohydrolases with distinct tissue distribution and homology to microbial arginine deiminases.. Biochem J.

[28] Dayoub H, Achan V, Adimoolam S, Jacobi J, Stuehlinger MC (2003). Dimethylarginine dimethylaminohydrolase regulates nitric oxide synthesis: genetic and physiological evidence.. Circulation.

[29] Sydow K, Mondon CE, Schrader J, Konishi H, Cooke JP (2008). Dimethylarginine dimethylaminohydrolase overexpression enhances insulin sensitivity.. Arterioscler Thromb Vasc Biol.

[30] Voight BF, Scott LJ, Steinthorsdottir V, Morris AP, Dina C (2010). Twelve type 2 diabetes susceptibility loci identified through large-scale association analysis.. Nat Genet.

[31] O'Dwyer MJ, Dempsey F, Crowley V, Kelleher DP, McManus R (2006). Septic shock is correlated with asymmetrical dimethyl arginine levels, which may be influenced by a polymorphism in the dimethylarginine dimethylaminohydrolase II gene: a prospective observational study.. Crit Care.

[32] Bai Y, Chen J, Sun K, Xin Y, Liu J (2009). Common genetic variation in DDAH2 is associated with intracerebral haemorrhage in a Chinese population: a multi-centre case-control study in China.. Clin Sci (Lond).

[33] Jones LC, Tran CTL, Leiper JM, Hingorani AD, Vallance P (2003). Common genetic variation in a basal promoter element alters DDAH2 expression in endothelial cells.. Biochem Biophys Res Commun.

[34] Andreozzi F, Laratta E, Procopio C, Hribal ML, Sciacqua A (2007). Interleukin-6 impairs the insulin signaling pathway promoting production of nitric oxide in human umbilical vein endothelial cells.. Mol Cell Biol.

[35] Andreozzi F, Formoso G, Prudente S, Hribal ML, Pandolfi A (2008). TRIB3 R84 variant is associated with impaired insulin mediated nitric oxide production in human endothelial cells.. Arterioscler Thromb Vasc Biol.

[36] Hribal ML, Presta I, Procopio T, Marini MA, Stančáková A (2011). ; on behalf of the EUGENE2 Consortium. Glucose tolerance, insulin sensitivity and insulin release in European non-diabetic carriers of a polymorphism upstream of CDKN2A and CDKN2B.. Diabetologia.

[37] Laakso M, Zilinskaite J, Hansen T, Welløv Boesgaard T, Vänttinen M (2008). for the EUGENE2 Consortium. Insulin sensitivity, insulin release and GLP-1 levels in subjects with IFG and/or IGT in the EUGENE2 study.. Diabetologia.

[38] Marini MA, Frontoni S, Mineo D, Bracaglia D, Cardellini M (2003). The Arg972 variant in insulin receptor substrate-1 is associated with an atherogenic profile in offspring of type 2 diabetic patients.. J Clin Endocrinol Metab.

[39] Cardellini M, Perego L, D'Adamo M, Marini MA, Procopio C (2005). C-174G polymorphism in the promoter of the interleukin-6 gene is associated with insulin resistance.. Diabetes Care.

[40] Stancáková A, Javorský M, Kuulasmaa T, Haffner SM, Kuusisto J (2009). Changes in insulin sensitivity and insulin release in relation to glycemia and glucose tolerance in 6,414 Finnish men.. Diabetes.

[41] Lorenzo C, Haffner SM, Stancáková A, Laakso M (2010). Relation of direct and surrogate measures of insulin resistance to cardiovascular risk factors in nondiabetic Finnish offspring of type 2 diabetic individuals.. J Clin Endocrinol Metab.

[42] Abhary S, Burdon KP, Kuot A, Javadiyan S, Whiting MJ (2010). Sequence variation in DDAH1 and DDAH2 genes is strongly and additively associated with serum ADMA concentrations in individuals with type 2 diabetes.. PLoS One.

[43] DeFronzo RA, Gunnarsson R, Bjorkman O, Olsson M, Wahren J (1985). Effects of insulin on peripheral and splanchnic glucose metabolism in noninsulin-dependent (type II) diabetes mellitus.. J Clin Invest.

[44] Laakso M, Edelman SV, Brechtel G, Baron AD (1990). Decreased effect of insulin to stimulate muscle blood flow in obese man: a novel mechanism for insulin resistance.. J Clin Invest.

[45] Abdul-Ghani MA, Matsuda M, Balas B, DeFronzo RA (2007). Muscle and liver insulin resistance indexes derived from the oral glucose tolerance test.. Diabetes Care.

